# *Schistosoma japonicum* Cystatin Alleviates Sepsis Through Activating Regulatory Macrophages

**DOI:** 10.3389/fcimb.2021.617461

**Published:** 2021-02-24

**Authors:** Hong Xie, Lingqin Wu, Xingzhi Chen, Shifang Gao, Huihui Li, Yuan Yuan, Jinbao Liang, Xiaoli Wang, Shuying Wang, Changyan Xu, Liang Chu, Bin Zhan, Rui Zhou, Xiaodi Yang

**Affiliations:** ^1^ Anhui Key Laboratory of Infection and Immunity of Bengbu Medical College, Bengbu, China; ^2^ Department of Basic Medical College, Bengbu Medical College, Bengbu, China; ^3^ Department of Pediatric, First Affiliated Hospital of Bengbu Medical College, Bengbu, China; ^4^ Department of General Surgery, Second Affiliated Hospital of Bengbu Medical College, Bengbu, China; ^5^ National School of Tropical Medicine, Baylor College of Medicine, Houston, TX, United States; ^6^ Anhui Province Key Laboratory of Immunology in Chronic Diseases of Bengbu Medical College, Bengbu, China

**Keywords:** cysteine protease inhibitor, *Schistosoma japonicum*, sepsis, macrophage, immunomodulation, adoptive transfer

## Abstract

Multi-organ failure caused by the inflammatory cytokine storm induced by severe infection is the major cause of death for sepsis. *Sj*-Cys is a cysteine protease inhibitor secreted by *Schistosoma japonicum* with strong immunomodulatory functions on host immune system. Our previous studies have shown that treatment with *Sj*-Cys recombinant protein (r*Sj*-Cys) attenuated inflammation caused by sepsis. However, the immunological mechanism underlying the immunomodulation of *Sj*-Cys for regulating inflammatory diseases is not yet known. In this study, we investigated the effect of *Sj*-Cys on the macrophage M2 polarization and subsequent therapeutic effect on sepsis. The r*Sj*-Cys was expressed in yeast *Pichia pastoris*. Incubation of mouse bone marrow-derived macrophages (BMDMs) with yeast-expressed r*Sj*-Cys significantly activated the polarization of macrophages to M2 subtype characterized by the expression of F4/80^+^ CD206^+^ with the elated secretion of IL-10 and TGF-β. Adoptive transfer of r*Sj*-Cys treated BMDMs to mice with sepsis induced by cecal ligation and puncture (CLP) significantly improved their survival rates and the systemic clinical manifestations of sepsis compared with mice receiving non-treated normal BMDMs. The therapeutic effect of *Sj*-Cys-induced M2 macrophages on sepsis was also reflected by the reduced pathological damages in organs of heart, lung, liver and kidney and reduced serological levels of tissue damage-related ALT, AST, BUN and Cr, associated with downregulated pro-inflammatory cytokines (IFN-gamma and IL-6) and upregulated regulatory anti-inflammatory cytokines (IL-10 and TGF-β). Our results demonstrated that *Sj*-Cys is a strong immunomodulatory protein with anti-inflammatory features through activating M2 macrophage polarization. The findings of this study suggested that *Sj*-Cys itself or *Sj*-Cys-induced M2 macrophages could be used as therapeutic agents in the treatment of sepsis or other inflammatory diseases.

## Introduction

Sepsis is a complex syndrome caused by a dysregulated host response to infection, leading to life-threatening organ dysfunction and failure (Singer et al., 2016). It remains a major cause of death throughout the world (Rudd et al., 2020; Yang et al., 2020b) regardless of appropriate antibiotic treatment and supportive care (Seymour et al., 2017; Williams et al., 2019; Baghdadi et al., 2020). Difficulties in developing drugs to treat sepsis also reflect the extreme complexity and variability of the serious condition (Fink and Shaw Warren, 2014; Huang et al., 2019). The progression of sepsis can be roughly categorized into two distinct but concomitant stages termed systemic inflammatory response syndrome (SIRS) and compensatory anti-inflammatory response syndrome (CARS) (Singer et al., 2016). SIRS is initiated by innate immune cells such as macrophages which release inflammatory cytokines upon the detection of pathogens or activation by LPS released by infected gram-negative bacteria, to mobilize host immune system to clean the infection (Ge et al., 2019; Huang et al., 2019). This stage is also referred to as the cytokine storm and is thought to be responsible for lethal organ damage during the early stage of sepsis (Guo et al., 2019; Sackett et al., 2019). On the other hand, CARS is a systemic deactivation of the immune system tasked with restoring homeostasis from an inflammatory state, which is related to the production of Th2 and regulatory cytokines such as IL-4, IL-10 and TGF-β (Ward et al., 2008). The timing and balance of SIRS and CARS responses have a powerful influence on clinical outcomes in sepsis.

Macrophages are common phagocytic cells for clearing invaded pathogens or apoptotic innate cells. The phenotype and function of macrophages may be polarized by microenvironment into M1-type (classically activated macrophage) or M2-type (alternatively activated macrophage). M1 macrophages are stimulated by IFN-gamma to promote inflammation by secreting pro-inflammatory cytokines IL-6, IL-12, and TNF-alpha. Th2 cells produced IL-4 can convert macrophages into M2-type that inhibit inflammation by secreting Arginase-I, IL-10 and TGF-β mainly involved in wound healing and tissue repair (Ley, 2017). Multiple studies have shown that M1 macrophages are associated with the SIRS stage of sepsis and are involved in the pathology and mortality in patients with sepsis. Thereby increasing M2 cells population and prolonging the presence of this macrophage subtype in the systemic microenvironment could be developed as a strategy to reduce organ damage and to increase tissue repair in sepsis condition (Xu et al., 2014; Liang et al., 2019; Takakura and Zandi-Nejad, 2019; Yang et al., 2019b; Jin et al., 2020). Additional studies also confirmed that M2 macrophages conferred a therapeutic effect on peritonitis-induced sepsis (Mehta et al., 2004) while M1-related inflammatory factor level was associated with the mortality of sepsis (Bozza et al., 2007).

Extensive experimental and epidemiological evidence suggest that helminth infections or helminth-derived products effectively modulate host immune responses to reduce deleterious inflammatory immune responses and stimulate regulatory responses, thereby exerting a therapeutic effect on inflammatory diseases (Ziegler et al., 2015; Chen et al., 2016; Jang et al., 2017; Jiang et al., 2018; Xu et al., 2018; Jin et al., 2019; Ryan et al., 2020). It has been observed that chronic infection of *Schistosoma japonicum* promoted macrophages from M1 to M2 polarization (Zhu et al., 2014; Giri and Cheng, 2019) and had a protective effect on sepsis (Du et al., 2011a). This protective effect on sepsis could be replicated in mice adoptively transferred with ex vivo programmed M2 macrophages (Du et al., 2011). The further study identified that *S. japonicum* soluble egg antigen (SEA) had the similar effect as *S. japonicum* infection to boost M2 polarization through STAT6 and PI3K pathway (Du et al., 2011). However, although the concept of worm therapy has been described as safe and effective, the application of living parasites or the derived raw materials still bears the risk of safety and side effects (Togre et al., 2018). Thus, therapeutic intervention by applying defined helminth-secreted protein with immunomodulation functions should be more practical and feasible to treat inflammatory diseases. It was found that *S. japonicum* adult worm secreted cysteine protease inhibitor or cystatin (*Sj*-Cys) played a significant role in immunomodulation of host immune system to reduce inflammatory responses as a survival strategy for the fluke living inside host (Chen et al., 2017), and has been successfully used to treat inflammatory bowel diseases (Wang et al., 2016b; Bisht et al., 2019), and collagen-induced arthritis (Liu et al., 2016) in mouse models. Our previous studies have identified that treatment with *Sj*-Cys significantly reduced the pathology caused by LPS-induced (Wan et al., 2018) or bacterial infestation-induced (Li et al., 2017) sepsis in mice with less inflammation and tissue damage through stimulating anti-inflammatory cytokines and inhibiting Th1 pro-inflammatory cytokines. In particular, treatment with *Sj*-Cys significantly reduced sepsis-induced cardiomyopathy (Gao et al., 2020). However, the immunological mechanism and targeted immune cells underlying the immunomodulation and therapeutic effect of *Sj*-Cys on sepsis remains unknown. Due to the important role of M2 macrophages in maintaining immune homeostasis from an inflammatory state to tissue repair, we would like to investigate whether treatment with *Sj*-Cys induces M2 macrophage polarization and whether *Sj*-Cys induced M2 polarization is involved in the therapeutic mechanism on sepsis. In this study, we induced sepsis with cecal ligation and puncture (CLP) in a mouse model. The mice with CLP-induced sepsis were adaptively transferred with *in vitro Sj*-Cys-induced M2 macrophages. We successfully determined that *Sj*-Cys stimulated bone marrow-derived macrophages (BMDMs) to differentiate to M2 and *Sj*-Cys-induced M2 macrophages had significant therapeutic effect on sepsis in adoptively transferred mice characterized with less tissue damage, lower pro-inflammatory cytokines and higher regulatory cytokines compared to mice transferred with normal BMDMs, indicating the M2 macrophage polarization is an important mechanism for the therapeutic effect of *Sj*-Cys on sepsis and other inflammatory diseases.

## Materials and Methods

### Expression and Purification of Recombinant *Sj*-Cys Protein (r*Sj*-Cys)

DNA encoding the full-length *Sj*-Cys (GenBank accession# FJ617450) was synthesized by Zoobio Biotechnology, China, and then subcloned into yeast expression vector pPIC9k using EcoRI and NotI sites. The correct insert and reading frame of the constructed recombinant plasmid *Sj*-Cys/pPIC9k was confirmed by double-stranded DNA sequencing. The plasmid *Sj*-Cys/pPIC9k was linearized with SacI and then transformed into *P. pastoris* GS115 by electroporation. The expression of r*Sj*-Cys with His-tag at C-terminus was induced with 0.5% methanol for 120 h. The expressed r*Sj*-Cys secreted in the medium was purified with immobilized metal affinity chromatography (IMAC) using a nickel column (Thermo, USA) as previously described (Zhan et al., 2005). The concentration of purified r*Sj*-Cys was measured using an enhanced BCA Protein Assay Kit (Beyotime, China). The purity of r*Sj*-Cys was measured with SDS-PAGE and the His-tag protein was confirmed by Western blotting with the anti-His antibody.

### Animals

The specific-pathogen-free male BALB/c mice with weight of 18-20 g, were purchased from the Animal Center of Anhui Medical University. All animal study protocols and procedures were reviewed and approved by the Animal Care and Use Committee of Bengbu Medical College and complied with the National Institutes of Health Guidelines for the Care and Use of Experimental Animals. All efforts were made to minimize the suffering of animals.

### Murine Model of Sepsis

A clinically relevant rodent model of sepsis was created by CLP as previously described (Li et al., 2017). Briefly, mice were anesthetized by intraperitoneal injection of 200 μL of 4% chloral hydrate (MACKLIN, China). The abdominal cavity was opened with a midline incision. The cecum was isolated, ligated (1.0 cm from the apex), and punctured with a 22-gauge needle, then returned to the abdominal cavity. The opened abdominal cavity was closed with sutures.

### Induction of Mouse Bone Marrow-Derived Macrophages (BMDMs)

Bone marrow cells were collected from sacrificed donor mice by flushing bone marrow cavities of femurs and tibias with complete DMEM medium (HyClone, USA) containing 10% fetal bovine serum (FBS) (EVERY GREEN, China) and penicillin (100 U/ml)/streptomycin (100 µg/ml) (Beyotime, China). The collected bone marrow cells were seeded in a 100 mm Petri dish and incubated with complete DMEM at 37°C, 5% CO_2_ for 4 h to collect adherent cells. The collected adherent cells were continuously co-cultured with murine macrophage colony-stimulating factor (M-CSF) (R&D Systems, USA) at 20 ng/ml for 7 days to stimulate the maturation of macrophages. Seven days after the culture, the matured bone marrow-derived macrophages (BMDMs) were examined using FITC-conjugated rat anti-mouse F4/80 (BioLegend, USA) and APC-conjugated rat anti-mouse CD11b (BioLegend, USA) staining.

### Macrophage Polarization

To determine the effect of r*Sj*-Cys on the macrophage polarization, a total of 1×10^6^ BMDMs obtained above were incubated with r*Sj*-Cys (2 ug/ml). The same number of BMDMs were incubated with LPS (100 ng/ml) (Solarbio, China) as M1 polarization control, and with IL-4 (10 ng/ml) + IL-10 (10 ng/ml) (R&D Systems, USA) as M2 polarization control. After being incubated for 24 h, cells from each group were measured for M1 marker (CD86) and M2 marker (CD206) by flow cytometry. For flow cytometry assay, BMDMs were fixed with fixable viability dye efluor 510 (BioLegend, USA) first in the dark for 10 min at RT to differentiate live/dead cells. After being washed with 2 ml PBS containing 1% FBS, the cells were collected by centrifuging at 500×g for 5 min at RT. After Fc receptors being blocked with α-CD16/32 (BioLegend, USA) for 10 min at RT, the cells were stained with FITC-conjugated rat anti-mouse F4/80 (BioLegend, USA) and APC-conjugated rat anti-mouse CD86 (Thermo Fisher Scientific, USA) for 30 min at 4°C. The cells were fixed and permeabilized using a Thermo Fixation/Permeabilization Kit (Thermo Fisher Scientific, USA) as per manufacturer’s instructions, then stained with PE-conjugated rat anti-mouse CD206 (BioLegend, USA) for 30 min at 4°C. The isotype-matched immunoglobulins (BioLegend, USA; Thermo Fisher Scientific, USA) and FMO were used as control for non-specific staining as baseline. The flow cytometry was performed with a flow cytometer DxP Athena (CYTEK, USA) and the data were analyzed using FlowJo-V10 software (BD Biosciences, USA).

### Adoptive Transfer of r*Sj*-Cys-Treated BMDMs to Mice With CLP-Induced Sepsis

A total of 56 mice were given CLP surgery to induce sepsis, then randomly divided into 4 groups; another 14 mice served as the blank control group. Thirty minutes after surgery, one group of 14 mice received intravenously with 1×10^6^ BMDMs treated with r*Sj*-Cys. Other three groups with the same number of mice received BMDMs treated with LPS, or LPS + r*Sj*-Cys, or PBS, respectively, as controls. Four mice from each group were euthanized 12 h after the macrophage transfer, blood was collected from each euthanized mouse and sera were obtained for serological tests, heart, lung, liver and kidney tissues were collected for histopathologic analysis. The survival rate was observed for 72 h for the left 10 mice from each group.

### Serological Test

The levels of alanine transaminase (ALT), aspartate transaminase (AST), blood urea nitrogen (BUN) and creatinine (Cr) in sera were used as biomarkers for tissue damage, cell disruption or failed functions of tissues including liver and kidney (Tesch, 2010; Kwo et al., 2017) in mice with sepsis. These biomarkers were measured in sera of experimental mice (4 of each group) by automatic chemistry analyzer (Beckman Coulter, USA) to evaluate sepsis-caused tissue injury.

### Cytokine Measurement

The concentrations of pro-inflammatory (IFN-gamma and IL-6) and regulatory (IL-10 and TGF-β) cytokines in the culture supernatants of BMDMs incubated with r*Sj*-Cys and other controls or in the sera collected from mice 12 h after macrophage transfer were determined using specific ELISA detection kits (Mouse IFN-gamma ELISA Kit, Mouse IL-6 ELISA Kit and Mouse IL-10 ELISA Kit from Dakewe Biotech, China and Mouse TGF-beta 1 ELISA Kit from ABclonal, USA) according to the manufacturer’s procedures. In order to remove the exogenously added IL-10 in the culture medium, the culture supernatant in the group with added IL-10 was removed after 24 h of culture, and cells were washed with PBS 3 times. The culture was continued with medium without IL-10 for another 24 h before harvested for IL-10 measurement.

### Histopathologic Analysis

Mouse heart, lung, liver and kidney were collected from four mice in each group euthanized 12 h after sepsis induction and receiving treated BMDMs. These tissues were fixed with 4% paraformaldehyde for 24 h, embedded in paraffin, sectioned to a thickness of 4 µm and stained with hematoxylin and eosin. Histological pathology was scored using a semi-quantitative scale as previously described. Briefly, the severity of heart damage was scored 0–4 as follows: 0 = normal; 1 = moderate (normal arrangement of myocardial fibers, punctate myocardial cell edema, degeneration, and necrosis); 2 = severe (normal arrangement of myocardial fibers, scattered myocardial cell edema, degeneration, and necrosis); 3 = extremely severe (loose arrangement of myocardial fibers, sheet-like myocardial cell edema, degeneration, and necrosis); and 4 = critical (loose arrangement of muscle fibers, breakage, and dissolution of myocardial fibers, diffuse edema, degeneration and necrosis of myocardial cells) (Li et al., 2017). Lung injury was determined by the alveolar congestion, tissue hemorrhage, inflammatory cell infiltration and scored 0–4 as follows: 0 = no pathology; 1 = mild (< 25% lung involvement); 2 = moderate (25–50% lung involvement); 3 = severe (50–75% lung involvement); 4 = extremely severe (> 75% lung involvement) (Yang et al., 2019a). Liver injury was determined as hepatocyte edema and tissue congestion/hemorrhage, inflammatory cell infiltration and scored as 0 = no pathology; 1 = mild (< 25% liver involvement); 2 = moderate (25–50% liver involvement); 3 = severe (50–75% liver involvement); 4 = very severe (> 75% lung involvement) (Wang et al., 2019). The degree of kidney injury was scored as follows: 0 = no pathology; 1 = mild (areas of tubular epithelial cell swelling, vacuolar degeneration, necrosis and desquamation involving < 25% of cortical tubules); 2 = moderate with tissue damaged involved > 25% but < 50%; 3 = severe (similar changes involving > 50% but < 75% of cortical tubules); 4 = extremely severe (similar changes involving > 75% of cortical tubules) (Li et al., 2019).

### Statistical Analysis

Statistical analysis was performed using SPSS 26.0 software (Chicago, USA). Data were expressed as mean ± SEM. Data with normal distribution and uniform variance were analyzed using unpaired, two-tailed Student’s *t*-test with Bonferroni adjustment, or ANOVA for multiple comparisons. A *P*-value less than 0.05 was considered as statistically significant.

## Results

### Expression of r*Sj*-Cys in Yeast

**Figure 1:**
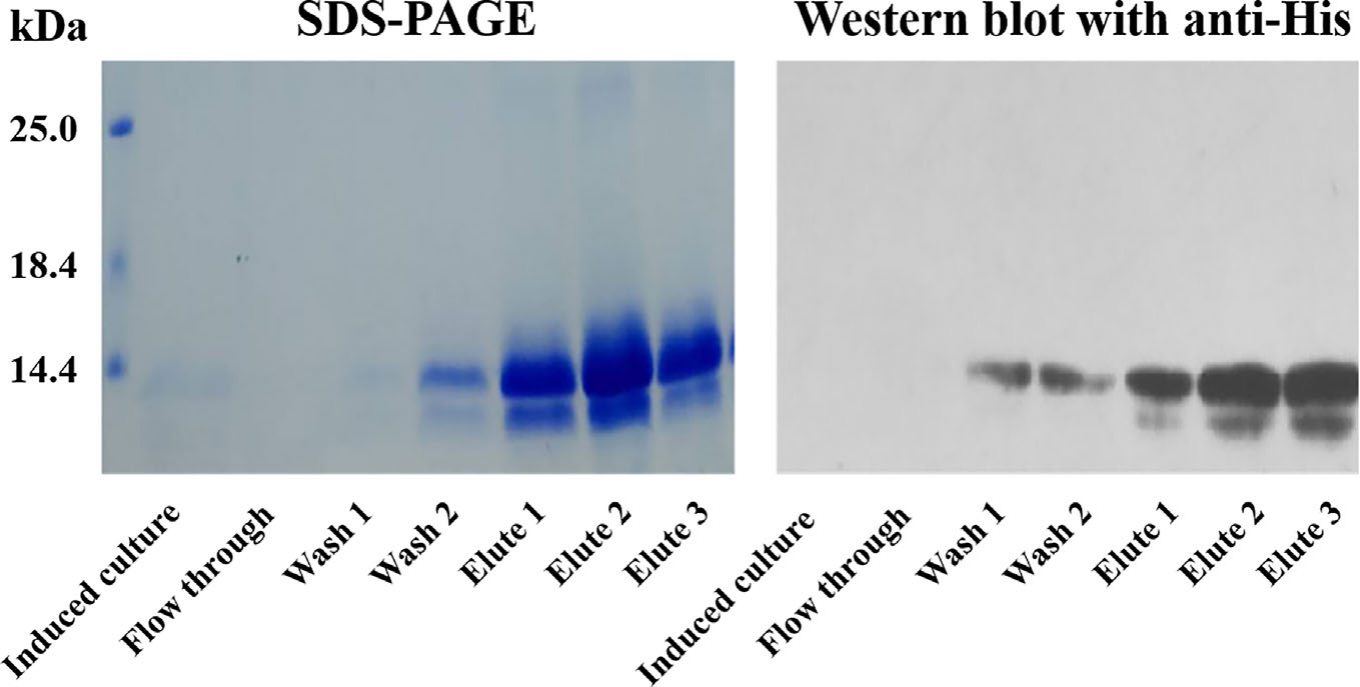
IMAC purification of recombinant *Sj-*Cys expressed in *P. pastoris* GS115. The r*Sj*-Cys with His-tag at C-terminus was expressed as a soluble protein in the culture medium. After binding on the nickel column, r*Sj*-Cys was eluted in a buffer containing imidazole. The purified protein was recognized by the anti-His antibody.

The r*Sj*-Cys with His-tag at C-terminus was successfully expressed as a soluble protein in *P. pastoris* GS115 under induction with 0.5% methanol for 120 h and purified with IMAC using a nickel column. The purified r*Sj*-Cys migrated as about 12 kDa on SDS-PAGE, the similar size as predicted by sequence (12.4 kDa). A small portion of degradation was observed at lower band (~11 kDa). The purified His-tagged r*Sj*-Cys was recognized by the anti-His antibody on Western blot (**Figure 1**). The smaller band was also recognized by the anti-His antibody, indicating it is a recombinant protein-derived product.

### r*Sj-*Cys Induced M2 Macrophage Polarization

**Figure 2:**
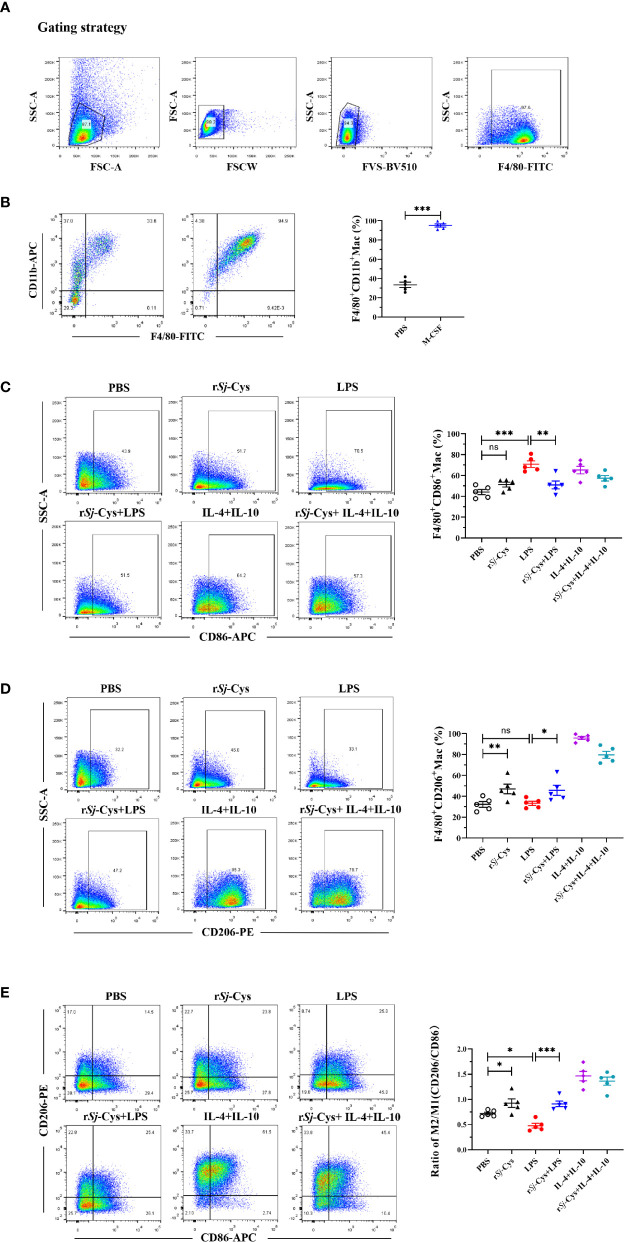
r*Sj*-Cys induced M2 macrophage polarization and reduced LPS-induced M1 macrophages phenotype *in vitro*. **(A)** The flow cytometry experiments were repeated by adding live/dead cell staining, and re-gated to differentiate dead cells, adhere cells and to block Fc. **(B)** BMDMs were obtained by incubating mouse bone marrow cells (adherent) with M-CSF for 7 days. The mature BMDMs were defined as CD11b^+^F4/80^+^ subpopulations using FACS. **(C–E)** BMDMs were incubated with r*Sj*-Cys (2 ug/ml), LPS (100 ng/ml), IL-4 (10 ng/ml) + IL-10 (10 ng/ml), r*Sj*-Cys + LPS, r*Sj*-Cys + IL-4 + IL-10, or PBS, respectively, for 24 h. The M1 (CD86) and M2 (CD206) markers were detected using FACS. n = 5. Data are expressed as mean ± SEM, ns, not significant, *P < 0.05, **P < 0.01, ***P < 0.001, ****P < 0.0001.

BMDMs were obtained by co-incubating with M-CSF (20 ng/ml) for 7 days. Flow cytometry measurement with a complete gating strategy (**Figure 2A**) to differentiate dead cells and adhere cells confirmed that more than 95% cells were labeled with CD11b^+^F4/80^+^, indicating most of the mouse bone marrow cells have been converted to BMDM cells (**Figure 2B**).

After being incubated with r*Sj-*Cys at 2 ug/ml for 24 h, 47.08 ± 4.59% BMDMs expressed CD206 which is significantly higher than BMDMs incubated with PBS (32.30 ± 2.72%) or LPS (33.32 ± 1.99%), however, incubation with r*Sj*-Cys did not affect the expression of CD86 on BMDMs compared with PBS control, indicating r*Sj*-Cys significantly stimulated M2 macrophage polarization, but not M1. In the control groups, M1 polarization (CD86) was strongly induced by LPS (100 ng/ml), and the IL-4 + IL-10 (each 10 ng/ml) induced M2 polarization (CD206). Interestingly, r*Sj*-Cys itself not only induced macrophage M2 polarization, but also significantly inhibited LPS-induced M1 polarization, however, the r*Sj*-Cys-induced M2 polarization was not affected in the presence of LPS (**Figures 2C–E**).

**Figure 3:**
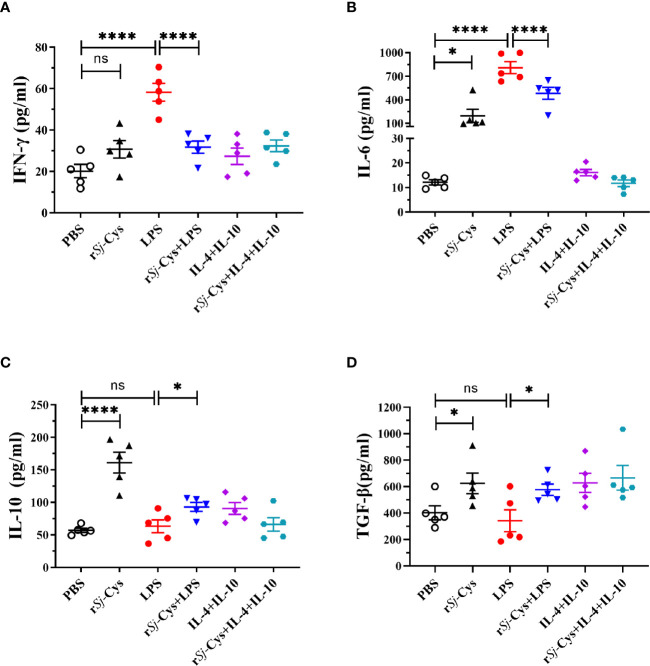
r*Sj*-Cys induced BMDMs to secrete M2 macrophage-related cytokines IL-10 and TGF-β. Mature BMDMs were stimulated with PBS, r*Sj*-Cys (2 ug/ml), LPS (100 ng/ml), IL-4 (10 ng/ml) + IL-10 (10 ng/ml), r*Sj*-Cys + LPS, or r*Sj*-Cys + IL-4 + IL-10 for 24 h. The levels of M2-related cytokines IL-10 **(C)**, TGF-β **(D)**, and M1-related cytokines IFN-gamma **(A)**, IL-6 **(B)** were measured in the supernatant by ELISA. n = 5. The results are presented as mean ± SEM, ns, not significant, *P < 0.05, **P < 0.01, ***P < 0.001, ****P < 0.0001.

The cytokine profiles in the culture supernatants of each incubation group also showed that r*Sj*-Cys induced BMDMs to secrete M2 macrophages-related IL-10 and TGF-β, and LPS was not able to inhibit r*Sj*-Cys induced M2-related cytokines (IL-10 and TGF-β). Incubation of BMDMs with r*Sj*-Cys also induced M1-related cytokines IFN-gamma and IL-6 compared to PBS control, however, co-incubation with r*Sj*-Cys reduced LPS-induced M1 cytokines (IFN-gamma and IL-6) (**Figure 3**).

### Adoptive Transfer of r*Sj*-Cys Treated-BMDMs Mitigated CLP-Induced Sepsis in Mouse

**Figure 4:**
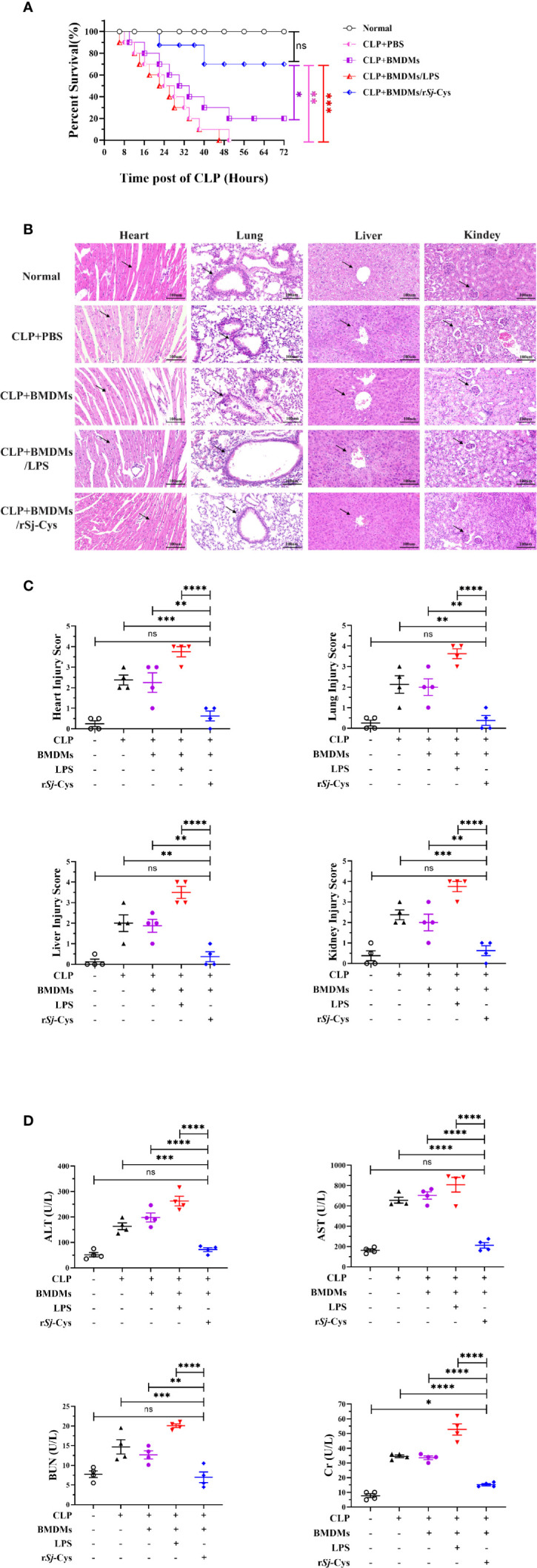
Adoptive transfer of rSj-Cys treated-BMDMs mitigated pathology caused by CLP-induced sepsis. **(A)** Mice adoptively transferred with r*Sj*-Cys-treated BMDMs significantly increased their survival rate up to 72 h (80%) compared to mice receiving PBS (0%), LPS-treated BMDMs (0%), or untreated BMDMs (20%) during the same observation period (n = 10). **(B, C)** The results of histopathology of heart, lung, liver and kidney stained with H&E staining from mice 12 h after CLP and transferred with BMDMs treated with r*Sj*-Cys, LPS or untreated BMDMs (n = 4). The pathological score comparison was shown on the right. The magnification ×400, scale bar = 100 µm. **(D)** The levels of ALT, AST, BUN, and Cr were measured in the sera from mice 12 h after receiving CLP and differently treated BMDMs (n = 4). The results are presented as mean ± SEM. ns, not significant, *P < 0.05, **P < 0.01, ***P < 0.001, ****P < 0.0001.

To evaluate the therapeutic effect of r*Sj*-Cys treated-BMDMs on sepsis, mice with CLP-induced sepsis were adoptively transferred with r*Sj*-Cys treated-BMDMs, the survival rate in each group was observed and the pathological improvement was identified in tissues of the treated mouse. All mice with CLP-induced sepsis without macrophage transfer or receiving LPS-treated BMDMs died within 24 h, however, 80% of mice adoptively transferred with rSj-Cys treated BMDMs survived up to 72 h while only 20% of mice receiving non-treated BMDMs survived (**Figure 4A**).

Histological examination showed significant damage in the hearts, lungs, livers, and kidneys of mice with CLP-induced sepsis. Specifically, all tissues showed varying degree of edema and swelling, inflammatory cell infiltration, disrupted or disordered tissue structure, hemorrhages, and congestion (**Figures 4B, C**). The amount of ALT, AST, BUN and Cr also remained at high levels in the sera of mice with sepsis (**Figure 4D**), further indicating the tissue damage caused by serious infection and sepsis.

After being passively transferred with r*Sj*-Cys-treated BMDMs, the mice with sepsis revealed significantly reduced tissue damage and inflammatory cell infiltration in all tissues of heart, lung, liver and kidney compared with tissues of mice receiving untreated-BMDMs or PBS only. Strikingly, sepsis mice receiving LPS-treated BMDMs showed significantly more serious damage in all tissues compared with mice receiving untreated BMDMs or PBS control groups (**Figures 4B, C**). However, the LPS-treated BMDMs exacerbated pathology was significantly reduced in all tissues of mice when receiving LPS-treated BMDMs co-incubated with r*Sj*-Cys, indicating *Sj*-Cys mitigated LPS-induced inflammation and tissue damage. The levels of ALT, AST, BUN, and Cr in serum were also greatly decreased in mice after receiving r*Sj*-Cys-treated BMDMs compared to mice receiving untreated BMDMs or PBS only, reflecting the reduced injury or damage of tissue cells (**Figure 4D**).

### r*Sj*-Cys Treated-BMDMs Downregulated Pro-Inflammatory Cytokines and Upregulated Regulatory Cytokines in Mice With Sepsis

**Figure 5:**
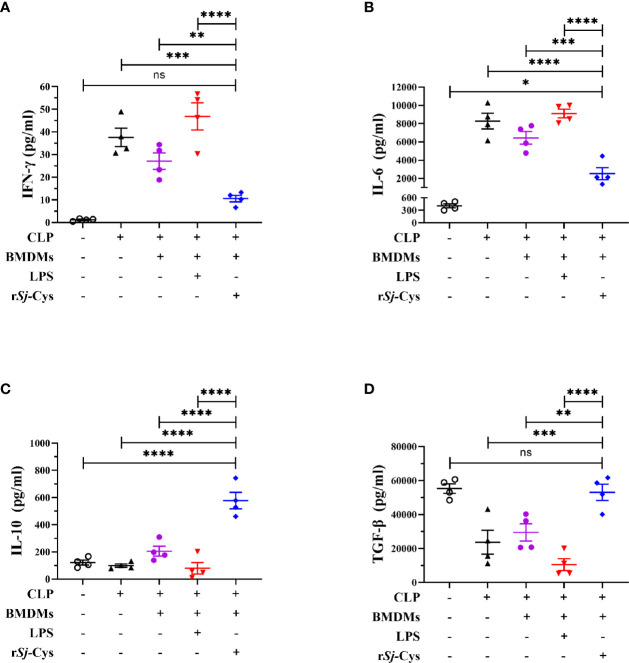
r*Sj*-Cys treated-BMDMs downregulated pro-inflammatory cytokines and upregulated regulatory cytokines in mice with sepsis. The levels of IFN-gamma **(A)**, IL-6 **(B)**, IL-10 **(C)**, and TGF-β **(D)** were measured in the sera of mice in each group using ELISA. n = 4. The results are presented as mean ± SEM. ns, not significant, *P < 0.05, **P < 0.01, ***P < 0.001, ****P < 0.0001.

To further understand the potential immunological mechanism underlying the therapeutic effect of r*Sj*-Cys treated BMDMs on sepsis in mice, the cytokine profile was measured in the serum of each treated mouse. As shown in **Figure 5**, the levels of inflammatory cytokines IFN-gamma and IL-6 were significantly reduced in mice with CLP-induced sepsis when receiving r*Sj*-Cys-treated BMDMs compared with sepsis mice receiving untreated BMDMs or PBS control. The mice receiving LPS-treated BMDMs showed even high production of IFN-gamma and IL-6 than the mice receiving non-treated BMDMs. On the other hand, the levels of regulatory cytokines IL-10 and TGF-β were significantly increased in sera of sepsis mice receiving r*Sj*-Cys-treated BMDMs compared with control mice receiving untreated or LPS-treated BMDMs or PBS only. The results further suggested that r*Sj*-Cys promoted M2 macrophage polarization which upregulated regulatory cytokines and downregulated pro-inflammatory cytokines production in donor mice, thus coffering a therapeutic effect on sepsis-induced inflammation and pathology.

## Discussion

In this article, we described a regulatory macrophage induced by the helminth immunomodulatory protein *Sj*-Cys and demonstrated its ability to efficiently suppress inflammatory responses in experimental CLP-induced sepsis. More and more evidence has shown that helminth infection causes damage on host, at meanwhile, it plays important roles in modulating host immune responses through secreting some proteins with immunomodulatory functions to reduce inflammation as a survival strategy (Venugopal et al., 2017; Ding et al., 2020). As a bystander effect, helminth infection or helminth-derived proteins enable to reduce host hypersensitivity to some allergens or autoantigen, therefore have been used to treat inflammatory diseases such as allergic asthma (Park et al., 2011; Aranzamendi et al., 2013; Ziegler et al., 2015; Sun et al., 2019a) or inflammatory bowel diseases (Du et al., 2011b; Ziegler et al., 2015; Sotillo et al., 2017; Xu et al., 2019). The identified mechanisms for helminth-induced immunomodulation are usually related to induce host Th2 and regulatory T cell (Treg) responses so as to reduce pro-inflammatory cytokines and subsequent inflammation (Shevach, 2009; Maruyama et al., 2011; Velavan and Ojurongbe, 2011; Gao et al., 2012; Kobpornchai et al., 2020).

Recent studies indicated that innate immune cells are involved in immunomodulation mediated by parasitic worms (Chen et al., 2016; Jiang et al., 2018; Jin et al., 2019; Cai et al., 2020), but little is known about the specific immune cells targeted by helminth immunomodulatory proteins or the mechanisms conferring suppression of ongoing inflammatory immune responses (Chuah et al., 2014). Macrophage cells are not only involved in the direct process of specific immune responses as antigen presenting cells, but also act as innate immune cells to clear pathogens or senescent/apoptotic cells through phagocytosis (Mosser and Edwards, 2008). In recent year, macrophages have been identified to play important roles in maintaining immune homeostasis by regulating the polarization of M1 or M2 subtype macrophages. The M1 macrophages stimulate inflammation by secreting pro-inflammatory cytokines and chemokines to promote clearing of invaded pathogens, while M2 reduce inflammation by secreting anti-inflammatory cytokines to play important roles in immunosuppressive function, wound healing and tissue repair (Wynn et al., 2013; Francos-Quijorna et al., 2016).

In our previous studies, we demonstrated that *Sj*-Cys protein suppressed inflammation when applied to mice with sepsis induced by CLP operation in a mouse model (Wan et al., 2018). In this study, we showed that the r*Sj*-Cys-modulated regulatory macrophages are sufficient to replicate the anti-inflammatory effects of r*Sj*-Cys. To gain insight into the immunomodulatory properties of r*Sj*-Cys, the effect of *Sj*-Cys on the induction of different macrophage subpopulations (M1 and M2) has been explored in the study. Our results showed that incubation of r*Sj*-Cys with BMDMs significantly stimulated M2 macrophage polarization (47.08% expressed CD206, **Figures 2C–E**) associated with the secretion of regulatory cytokines IL-10 and TGF-β (**Figure 3**). Interestingly, incubation of r*Sj*-Cys with BMDMs even inhibited the secretion of LPS-induced pro-inflammatory cytokines IFN-gamma and IL-6, and the addition of LPS could not prevent r*Sj*-Cys-treated BMDMs from the secretion of IL-10 and TGF-β, (**Figure 3**), indicating r*Sj*-Cys is able to inhibit bacterial infection-induced inflammation. It is well known that IL-10 and TGF-β play an important role in establishing tolerance and suppression of inflammation diseases (Murray et al., 2014; Li et al., 2018; Yan et al., 2020). IL-10 is a particularly noteworthy cytokine because it has anti-inflammatory properties and influences in the activity of several cell types of the immune system, thereby regulating the immune network (Verma et al., 2016; Mazer et al., 2019; Saraiva et al., 2020). The TGF-β is a pleiotropic cytokine with important immunoregulatory functions to reduce activities related to immune disorders (Branchett and Lloyd, 2019). Results in this study confirm that *Sj*-Cys not only stimulates the polarization of M2 macrophage, but also inhibits macrophage’s pro-inflammatory responses to LPS, further indicating the ability of r*Sj*-Cys to stimulate M2 macrophage regulatory property and reverse LPS-induced M1 differentiation and inflammation caused by sepsis or other bacterial infection.

To verify whether r*Sj*-Cys-induced M2 macrophages are involved in the therapeutic effect of r*Sj*-Cys on sepsis, the mice with CLP-induced sepsis were adoptively transferred with r*Sj*-Cys-treated BMDMs. The results revealed that the 72 h survival rate of mice with CLP-induced sepsis was significantly improved after being transferred with r*Sj*-Cys-treated BMDMs (80%) compared with mice receiving non-treated normal BMDMs (20%) or LPS-treated BMDMs (mimicking sepsis condition) or PBS only (0%) (**Figure 4A**), confirming that the therapeutic effect of *Sj*-Cys can be conducted by *Sj*-Cys-induced M2 macrophages. The therapeutic effect of *Sj*-Cys-induced M2 macrophages on sepsis was also reflected by the reduced pathological damages in organs of heart, lung, liver and kidney caused by sepsis (**Figures 4B, C**) and reduced serological levels of tissue injury-related enzymes or proteins including ALT, AST, BUN and Cr (**Figure 4D**). The cytokine profile in sera of mice receiving *Sj*-Cys-induced M2 macrophages also showed significantly upregulated anti-inflammatory IL-10 and TGF-β and down-regulated pro-inflammatory IFN-gamma and IL-6. This inhibitory cytokine profile could be derived from the direct secretion of transferred M2 macrophages or subsequent inhibitory immune responses induced by transferred M2 subtype in recipient mice. All mice with sepsis adoptively transferred with LPS-activated BMDMs demonstrated more severe inflammation and tissue damage, aggravating the bacterial infection caused inflammation. Our results are consistent with the previous finding that systemic inflammation of sepsis leads to partial activation of BMDM, deteriorating the inflammation and pathological damage (Zhu et al., 2014).

All results in this study provide the evidence at the first time that r*Sj*-Cys-induced M2 macrophage polarization was involved in the therapeutic effects of r*Sj*-Cys on sepsis-caused inflammation and tissue damages, providing another immunological mechanism pathway for immunomodulatory functions of helminth-secreted proteins except for the Treg regulatory pathway. However, the M2 macrophage-secreted regulatory cytokines IL-10 and TGF-β could further stimulate Treg responses (Wang et al., 2016a). It is also identified that helminth-secreted proteins stimulated Treg response through up-regulating PD-1 in CD4^+^ T cells (Cheng et al., 2018). A recent study also showed that helminth-derived proteins stimulated Treg differentiation through activating dendritic cells (Sun et al., 2019b). It is unknown if PD-1 or dendritic cells pathways are involved in the r*Sj*-Cys-initiated immunomodulation.

The finding of the therapeutic effect of r*Sj*-Cys-induced M2 on the inflammatory sepsis in this study provides evidence that a therapeutic approach for the treatment of inflammatory disorders based on cells modulated by a single parasitic molecule may be feasible. Such an approach would be attractive because it allows the exploitation of helminth immunomodulatory therapy without the risk of living worms or the side effects of direct application of worm-derived proteins. Cell-based therapies constitute a promising approach in which cells are differentiated into an immunosuppressive or regulatory phenotype in administered patients (Ledesma-Soto et al., 2015; Kang et al., 2019). Several basic and clinical immunotherapy studies have been performed with adoptive cells, such as T cells, NK cells, dendritic cells, to targeting cancer (Magalhaes et al., 2019; Wculek et al., 2019; Xiao et al., 2019). Cell immunotherapy studies advance not only in the field of cancer but also in inflammatory disease. Adoptive transfer of helminth protein induced M2 (Ziegler et al., 2015; Reyes et al., 2016) or SNX10 deficiency induced M2 macrophages (You et al., 2016) ameliorated inflammatory bowel diseases in wild-type mice. There is evidence that mesenchymal stem/stromal cells (MSCs) could be used as a therapy for sepsis and acute respiratory distress syndrome (Byrnes et al., 2021). Adoptive transfer of macrophages containing antimicrobial peptides was successfully used for the treatment of multidrug-resistant bacteria-induced sepsis in mice (Hou et al., 2020). Inhibition of mitophagy promoted macrophage activation favored bactericidal clearance, adoptive transfer of these macrophages to mice with sepsis resulted in better survival (Patoli et al., 2020). It was also showed that adoptive transfer of polarized M2c macrophages reduced acute rejection to liver transplantation in rat (Yang et al., 2020a). The macrophages have been evaluated as good candidates for cell-based therapeutic intervention not only for inflammatory diseases, but also for autoimmune diseases (De Dios Ruiz-Rosado et al., 2017).

Our results further confirmed the strong immunomodulatory functions of *Sj*-Cys, especially on the polarization of macrophages to regulatory M2 subtype. Adoptive transfer of *Sj*-Cys-induced M2 macrophages significantly mitigated the inflammation and tissue damage in mice with sepsis, providing a novel therapeutic approach for the treatment of inflammatory disorders.

## Data Availability Statement

The original contributions presented in the study are included in the article/supplementary material. Further inquiries can be directed to the corresponding authors.

## Ethics Statement

The animal study was reviewed and approved by The Animal Care and Use Committee of Bengbu Medical College.

## Author Contributions

XY, RZ, HX, and LW conceived and designed the study. HX, LW, SG, JL, SW, and CX performed the experiments. XC, HL, YY, XW, and LC analyzed the data. HX and LW wrote the manuscript. BZ, XY, and RZ critically revised the manuscript. All authors contributed to the article and approved the submitted version.

## Funding

This study was supported by the Science Foundation of Anhui Province (2008085MH260 and gxbjZD15); the Program of Natural Science Foundation of the Anhui Higher Education Institutions (KJ2019A0383, KJ2020A0554, KJ2020A0566 and KJ2020A0572); 512 Talents Development Project of Bengbu Medical College (by51201205 and by51201306); the Science Foundation of Bengbu Medical College (BYTM2019002, BYLK201818 and BYKY2019033ZD); the Postgraduate Scientific Research Innovation Program of Bengbu Medical College (Byycx1903) and the National University Students’ Innovation and Entrepreneurship Training Program (201910367002 and 202010367006).

## Conflict of Interest

The authors declare that the research was conducted in the absence of any commercial or financial relationships that could be construed as a potential conflict of interest.
